# Influenza vaccination motivators among healthcare personnel in a large acute care hospital in Israel

**DOI:** 10.1186/s13584-016-0112-5

**Published:** 2016-10-26

**Authors:** Amir Nutman, Naomi Yoeli

**Affiliations:** Tel-Aviv Sourasky Medical Center, 6 Weizmann St., Tel Aviv, 64239 Israel

**Keywords:** Influenza vaccine, Knowledge, perceptions and attitudes, Healthcare personnel vaccination uptake, Survey

## Abstract

**Background:**

Vaccinating healthcare personnel (HCP) against influenza is important to prevent transmission and morbidity among patients and staff.

**Methods:**

We conducted an online survey assessing knowledge, perceptions and attitudes concerning influenza vaccination among HCP. Multivariate logistic regression was performed to identify independent predictors of vaccination.

**Results:**

The survey was completed by 468 HCP representing all categories of staff. Doctors believed that vaccination was the best way to prevent influenza and perceived the vaccine less harmful as compared to nurses and allied health professionals. Getting vaccinated was associated with a greater likelihood of recommending vaccination to patients: 86 % vs. 54 % in vaccinated and unvaccinated HCP, respectively. Reasons for vaccine refusal were fear of needles (19 %); fear of side effects (66 %) and lack of time (16 %). In the multivariate analysis, survey items that were independently associated with vaccination were beliefs that: vaccine effectively prevents influenza (OR 4.07 95 % CI 2.51, 6.58); HCP are at increased risk of influenza (OR 2.82 95 % CI 1.56, 5.13); vaccine can cause influenza (OR 0.41 95 % CI 0.25, 0.65); contracting influenza is likely in the absence of vaccination (OR 1.96 95 % CI 1.12, 3.42); and that HCP might transmit influenza to their family (OR 4.54 95 % CI 1.38, 14.97). The belief that HCP might transmit influenza to patients was not independently associated with vaccine uptake.

**Conclusion:**

Our study revealed misconceptions and knowledge gaps concerning the risk of influenza and the influenza vaccine. There were significant differences in knowledge and attitudes between healthcare professions. HCP decline vaccination because they do not perceive a personal risk of influenza infection and are concerned about side effects. Thus, in order to increase vaccination rates it is important to educate HCP to correct misconceptions concerning vaccine efficacy and safety, while promoting the benefit of getting vaccinated in order to protect themselves and their families.

## Background

Healthcare personnel (HCP) are an important priority group for influenza vaccination in order to protect them against influenza infection and to prevent transmission to patients, especially high-risk populations such as immune-compromised and elderly patients [1–3]. Despite this, vaccination coverage of HCP is suboptimal worldwide [4, 5] and in the United States many institutions have successfully implemented a mandatory vaccination policy as a means to increase coverage [6].

In Israel, the Ministry of Health recommends influenza vaccination for all HCP and set a quality target of 60 % coverage [7]; however, there are no legal means to enforce this policy and vaccination uptake is unsatisfactory [8]. In the 2014–2015 influenza season, the average vaccination rate in acute care hospitals in Israel was 41 % (range 14–78 %): 47 % among doctors, 35 % among nurses and 37 % among allied health professionals [9].

As part of a program to increase vaccination coverage among HCP at our hospital, we conducted an online survey to assess motivating factors and barriers for immunization, identify target groups for intervention and explore attitudes toward a policy of mandatory vaccination.

## Methods

### Setting

The study was conducted at Tel-Aviv Sourasky Medical Center, a 1450-bed tertiary care academic hospital in Israel. Influenza vaccination is offered free of charge for all clinical and non-clinical personnel every year. In order to make vaccines more accessible, they are administered by mobile nurse teams who come to the clinical units, the personnel clinic and in public areas such as the cafeteria and conferences. Vaccines are also distributed to the wards for vaccinating staff working evening and night shifts. The influenza campaign also consists of educational material (posters, mailings and talks) and hospital leadership involvement.

### Data collection

Information about overall vaccination rate for the 2014–2015 influenza season was collected from employee immunization records.

The online survey was conducted at the end of the flu campaign of 2014–2015. Employees were invited to complete the online survey via an e-mail with a link to the survey. In order to increase participation, the survey was anonymous.

The survey consisted of 30 questions: demographic information including age, gender, profession (doctor, nurse, allied health professional or others including administrative and support staff), and department; knowledge about influenza disease and vaccine; perception of flu risk; perception of vaccine efficacy and side-effects; practices of recommending vaccination to family and patients, and attitude towards a mandatory vaccination policy. Each participant was asked to indicate whether they had been vaccinated in the survey year. Because the survey was anonymous, participant information could not be cross-referenced to immunization records. Participants who indicated that they were not vaccinated were asked about inhibiting factors: fear of vaccine side effects, fear of needles, lack of time and medical exemption from vaccination. Participants who indicated that they were vaccinated were asked about side effects after vaccination.

Answers to survey questions took two forms: agree, disagree or unsure and yes or no. Regarding recommending vaccination to patients, participants were allowed to answer “not relevant” if not engaged in direct patient care.

### Data analysis

We limited our analysis to participants who completed the entire survey. Data were summarized descriptively. For the purpose of analysis, “unsure” was grouped with “disagree”. Associations of survey items with profession were analyzed by chi-square tests. Odds ratios and 95 % confidence intervals for vaccination were calculated by simple logistic regression. Multivariate logistic regression, using forward selection of variables, was performed to identify independent predictors of vaccination among HCP. Demographic information and all survey questions were considered for the model. For all tests performed, a two-sided *p*-value ≤0.05 was considered statistically significant.

Data were analyzed using SPSS version 22 (IBM, Armonk, NY).

## Results

### Influenza vaccine uptake

During the 2014–2015 influenza season, the overall influenza vaccination rate at our hospital was 42 %: 56 % among physicians, 41 % among nurses, 37 % among allied health professionals, and 30 % among others (administration and support staff).

### Survey participants

The survey was completed by 468 employees. Demographic characteristics of participants are summarized in Table 1. Among survey participants, 310 (66 %) indicated they were vaccinated: 102 (84 %) of physicians, 60 (65 %) of nurses, 69 (57 %) of allied health professions and 79 (59 %) of others.

**Table 1:** Characteristics of study sample (*N* = 468)

Characteristic	*n* (%)
Sex female	339 (72.4)
Age category	
18–39 years	195 (41.7)
40–65 years	258 (55.1)
> 65 years	15 (3.2)
Profession	
Nurses	93 (19.9)
Doctors	121 (25.9)
Allied health professionals	121 (25.9)
Others^a^	133 (28.4)
Department	
Medical	150 (32.1)
Surgical	84 (17.9)
Obstetrics & Gynecology	15 (3.2)
Pediatrics	52 (11.1)
Radiology	19 (4.1)
Laboratories	32 (6.8)
Other Non-clinical	116 (24.8)
Vaccinated against influenza	310 (66.2)

^a^Other staff members including administrative and support staff

### Knowledge concerning influenza

Results of survey questions concerning knowledge about influenza are presented in Table 2: almost all participants agreed that influenza is widespread and can have severe complications including death. However, only 385 (82 %) of participants agreed that as HCP they are at increased risk for infection by influenza. This proportion was higher among doctors (94 %) than among nurses and allied health professionals (79 %). The OR for vaccination was 2.2 (95 % CI 1.1, 4.5) for participants who agreed that influenza can have severe complications, and 5.8 (95 % CI 3.5, 9.6) for participants who agreed that they are at increased risk for influenza.

**Table 2:** Knowledge concerning influenza disease (*N* = 468)

	All	Nurses	Doctors	Allied health professionals	Others	*p*-value^a^	OR for vaccination (95 % CI)
Influenza is widespread and can affect any person at any age							
Agree, *n* (%)	459 (98.1)	92 (98.9)	120 (99.2)	120 (99.2)	127 (95.5)	0.085	0.981 (0.242, 3.974)
Disagree, *n* (%)	1 (0.2)	1 (1.1)					Reference
Unsure, *n* (%)	8 (1.7)		1 (0.8)	1 (0.8)	6 (4.5)		
Influenza can have severe complications including death							
Agree, *n* (%)	435 (92.9)	86 (92.5)	120 (99.2)	110 (90.9)	119 (89.5)	0.015	2.215 (1.087, 4.513)
Disagree, *n* (%)	9 (1.9)	3 (3.2)		2 (1.7)	4 (3)		Reference
Unsure, *n* (%)	24 (5.1)	4 (4.3)	1 (0.8)	9 (7.4)	10 (7.5)		
Hospital workers are at increased risk of contracting influenza because of their job							
Agree, *n* (%)	385 (82.3)	73 (78.5)	114 (94.2)	96 (79.3)	102 (76.7)	0.001	5.755 (3.449, 9.602)
Disagree, *n* (%)	25 (5.3)	8 (8.6)	1 (0.8)	9 (7.4)	7 (5.3)		Reference
Unsure, *n* (%)	58 (12.4)	12 (12.9)	6 (5)	16 (13.2)	24 (18)		

^a^ *p*-value for association between survey item and profession

### Knowledge concerning influenza vaccine

Results of survey questions concerning knowledge about vaccine are presented in Table 3: 292 (62 %) of participants agreed that vaccination is the best way to prevent influenza. This proportion was higher among doctors (89 %) than among nurses (55 %) and allied health professionals (49 %). The OR for vaccination was 7.4 (95 % CI 4.8, 11.2) for participants who agreed that vaccination is the best way to prevent influenza. Almost half of participants, 228 (48.7 %), agreed that vaccination can cause influenza. This belief was more common among nurses (61 %) and allied health professionals (55 %) than among doctors (22 %). The OR for vaccination was 0.28 (95 % CI 0.19, 0.42) for participants who agreed that vaccine can cause the flu. Only 269 (57.5 %) disagreed that vaccine side effects can be more severe than the flu; disagreeing was less common among nurses (45 %) and allied health professionals (55 %) than among doctors (81 %). The OR for vaccination was 0.35 (95 % CI 0.22, 0.55) for participants who agreed that vaccine side effects can be more severe than the flu.

**Table 3:** Knowledge concerning influenza vaccine (*N* = 468)

	All	Nurses	Doctors	Allied health professionals	Others	*p*-value^a^	OR for vaccination (95 % CI)
The most effective way to prevent influenza is vaccination							
Agree, *n* (%)	292 (62.4)	51 (54.8)	108 (89.3)	59 (48.8)	74 (55.6)	<0.001	7.328 (4.778, 11.238)
Disagree, *n* (%)	60 (12.8)	19 (20.4)	3 (2.5)	17 (14)	21 (15.8)		Reference
Unsure, *n* (%)	116 (24.8)	23 (24.7)	10 (8.3)	45 (37.2)	38 (28.6)		
Vaccine side effects can be more severe than the flu							
Agree, *n* (%)	93 (19.9)	30 (32.3)	12 (9.9)	23 (19)	28 (21.1)	0.001	0.348 (0.219, 0.554)
Disagree, *n* (%)	269 (57.5)	42 (45.2)	98 (81)	67 (55.4)	62 (46.6)		Reference
Unsure, *n* (%)	106 (22.6)	21 (22.6)	11 (9.1)	31 (25.6)	43 (32.3)		
Vaccine can cause the flu							
Agree, *n* (%)	228 (48.7)	57 (61.3)	26 (21.5)	67 (55.4)	78 (58.6)	<0.001	0.280 (0.186, 0.421)
Disagree, *n* (%)	150 (32.1)	23 (24.7)	80 (66.1)	30 (24.8)	17 (12.8)		Reference
Unsure, *n* (%)	90 (19.2)	13 (14)	15 (12.4)	24 (19.8)	38 (28.6)		
Pregnant women should not get vaccinated							
Agree, *n* (%)	54 (11.5)	15 (16.1)	7 (5.8)	12 (9.9)	20 (15)	0.051	0.301 (0.168, 0.538)
Disagree, *n* (%)	226 (48.3)	42 (45.2)	89 (73.6)	61 (50.4)	34 (25.6)		Reference
Unsure, *n* (%)	188 (40.2)	36 (38.7)	25 (20.7)	48 (39.7)	79 (59.4)		
People who had the flu don’t need to get vaccinated							
Agree, *n* (%)	4 (0.9)	1 (1.1)	1 (0.8)	1 (0.8)	1 (0.8)	0.995	1.534 (0.158, 14.87)
Disagree, *n* (%)	439 (93.8)	88 (94.6)	117 (96.7)	113 (93.4)	121 (91)		Reference
Unsure, *n* (%)	25 (5.3)	4 (4.3)	3 (2.5)	7 (5.8)	11 (8.3)		

^a^ *p*-value for association between survey item and profession

### Perceived influenza risk

Results of survey questions concerning perceived personal risk are presented in Table 4: Only 75 (16 %) agreed that they get sick with the flu easily and only 144 (31 %) agreed that if they don’t get vaccinated they get sick with the flu. There was no significant difference between healthcare professions. The OR for vaccination was 1.6 (95 % CI 0.9, 2.8) for participants who agreed that they catch the flu easily, and 3.5 (95 % CI 2.2, 5.8) for participants who agreed that if they don’t get vaccinated they get sick with the flu.

**Table 4:** Perceived personal risk (*N* = 468)

	All	Nurses	Doctors	Allied health professionals	Others	*p*-value^a^	OR for vaccination (95 % CI)
I catch the flu easily							
Agree, *n* (%)	75 (16)	10 (10.8)	22 (18.2)	19 (15.7)	24 (18)	0.431	1.613 (0.921, 2.824)
Disagree, *n* (%)	292 (62.4)	59 (63.4)	78 (64.5)	80 (66.1)	75 (56.4)		Reference
Unsure, *n* (%)	101 (21.6)	24 (25.8)	21 (17.4)	22 (18.2)	34 (25.6)		
If I don’t get vaccinated I will get sick with the flu							
Agree, *n* (%)	144 (30.8)	28 (30.1)	47 (38.8)	35 (28.9)	34 (25.6)	0.132	3.526 (2.158, 5.761)
Disagree, *n* (%)	189 (40.4)	35 (37.6)	44 (36.4)	58 (47.9)	52 (39.1)		Reference
Unsure, *n* (%)	135 (28.8)	30 (32.3)	30 (24.8)	28 (23.1)	47 (35.3)		
If I get sick with the flu I may infect my family							
Agree, *n* (%)	446 (95.3)	91 (97.8)	118 (97.5)	115 (95)	122 (91.7)	0.09	7.355 (2.66, 20.333)
Disagree, *n* (%)	10 (2.1)	1 (1.1)	3 (2.5)	3 (2.5)	3 (2.3)		Reference
Unsure, *n* (%)	12 (2.6)	1 (1.1)		3 (2.5)	8 (6)		
If I get sick with the flu I may infect my patients							
Agree, *n* (%)	431 (92.1)	89 (95.7)	118 (97.5)	107 (88.4)	117 (88)	0.008	4.092 (2.022, 8.282)
Disagree, *n* (%)	15 (3.2)	2 (2.2)		7 (5.8)	6 (4.5)		Reference
Unsure, *n* (%)	22 (4.7)	2 (2.2)	3 (2.5)	7 (5.8)	10 (7.5)		

^a^ *p*-value for association between survey item and profession

Most of the participants (95 %) agreed that if they get sick with influenza they may infect their family. The proportion of participants who agreed that they may infect patients was slightly lower (92 %); agreement was more common among doctors (98 %) and nurses (96 %) than among allied health professionals (88 %). The OR for vaccination was 7.4 (95 % CI 2.7, 20.3) for participants who agreed that if they get sick with influenza they may infect their family, higher compared to the OR for vaccination (4.1, 95 % CI 2, 8.3) for participants who agreed that if they get sick with influenza they may infect patients.

### Practices of recommending vaccination and attitude towards mandatory vaccination

Practices regarding recommending vaccination are presented in Table 5: 322 (69 %) recommended vaccination to family, and 210 (77 % of participants engaged in direct patient care) indicated that they recommended vaccination to patients. Of the participants who were vaccinated, 86 % recommended vaccination to family and 86 % recommended vaccination to patients, as compared to 35 and 54 %, respectively, in participants who were unvaccinated.

**Table 5:** Practices regarding recommending vaccination (*N* = 468)

	All	Nurses	Doctors	Allied health professionals	Others	*p*-value^b^
Did you recommend vaccination to family?						
Yes, *n* (%)	322 (68.8)	61 (65.6)	105 (86.8)	75 (62)	81 (60.9)	<0.001
No, *n* (%)	146 (31.2)	32 (34.4)	16 (13.2)	46 (38)	52 (39.1)	
Did you recommend vaccination to patients?						
Yes, *n* (%)	210 (44.9)	58 (62.4)	101 (83.5)	26 (21.5)	25 (18.8)	<0.001
No, *n* (%)	64 (13.7)	13 (14)	9 (7.4)	29 (24)	13 (9.8)	
NR^a^, *n* (%)	194 (41.5)	22 (23.7)	11 (9.1)	66 (54.5)	95 (71.4)	
Are you in favor of mandatory vaccination policy for hospital workers?						
Yes, *n* (%)	249 (53.2)	52 (55.9)	68 (56.2)	57 (47.1)	72 (54.1)	0.464
No, *n* (%)	219 (46.8)	41 (44.1)	53 (43.8)	64 (52.9)	61 (45.9)	

^a^ *NR* Not relevant

^b^ *p*-value for association between survey item and profession

Only 249 (53 %) of participants were in favor of a mandatory vaccination policy: 65 % among vaccinated participants, and 30 % among unvaccinated participants.

### Reasons for vaccine refusal and vaccine side effects

Reasons for vaccine refusal in unvaccinated participants were: fear of needles (19 %); fear of side effects (66 %); lack of time (16 %); and medical exemption (4 %). Fear of side effects was the most frequent reason for all professions. Fear of needles was lower among doctors and nurses than other professions. Lack of time was more frequent for doctors than for other professions.

Among vaccinated participants, 27 % indicated they had side effects after receiving the vaccine; 38 % of nurses, 22 % of doctors, 26 % of allied health professionals, and 25 % of others.

### Multivariate analysis

Survey items that were independently associated with vaccination in the multivariate logistic regression model were: “The most effective way to prevent influenza is vaccination” (OR 4.07 95 % CI 2.51, 6.58); “Hospital workers are at increased risk of contracting influenza because of their job” (OR 2.82 95 % CI 1.56, 5.13); “Vaccine can cause the flu” (OR 0.41 95 % CI 0.25, 0.65); “If I don’t get vaccinated I will get sick with the flu” (OR 1.96 95 % CI 1.12, 3.42); and “If I get sick with the flu I may infect my family” (OR 4.54 95 % CI 1.38, 14.97). The survey item “If I get sick with the flu I may infect my patients” was not independently associated with vaccine uptake. Furthermore, gender, age, ward and profession were not significant predictors of vaccination.

## Discussion

Influenza vaccination coverage at our hospital is unsatisfactory: 42 % of all HCP. However, our vaccination coverage was consistent with the national average [9]. This study revealed that there are misconceptions and significant gaps in knowledge of the risk of influenza and of the influenza vaccine among HCP at our hospital, despite extensive efforts to educate HCP during the influenza vaccination campaign. HCPs’ knowledge of influenza vaccine has been directly correlated with vaccination uptake [10, 11]. In a meta-analysis of studies sponsored by the US Centers for Disease Control and Prevention, HCPs’ misconceptions about influenza vaccination were similar to those held by the general public [12]. Similarly, in a study by Gesser-Edelsburg et al. concerning Israeli HCP's attitudes towards the avian influenza vaccine in an outbreak setting, there was no difference between HCP and the general public regarding support for vaccination [13].

In a large survey of HCP in 2 US medical centers, the most frequent barriers to vaccination against influenza cited by participants who declined vaccination were fear of side effects (39 %) and fear of contracting influenza from the vaccine (25 %) [14]. In our study, almost half of participants agreed that you can get sick with influenza from being vaccinated and only 58 % disagreed that vaccine side effects are worse than influenza infections. Among participants who declined vaccination, fear of side effects was the most frequent reason (66 %), consistent with previous studies [15].

Our study also points to significant differences in knowledge and attitudes between healthcare professions. The proportion of doctors who agreed that vaccination was the best way to prevent influenza was higher as compared to nurses and allied health professionals. Similarly, the proportion of HCP who perceived the vaccine to be harmful was higher among nurses and allied health professionals than among doctors. Similar differences between physicians and nurses have been reported in previous studies [16]. This may explain the lower vaccination rates of nurses and allied health professionals as compared to doctors seen in our study and also at the national level [9].

Other barriers to vaccination reported in previous studies include a low perceived vaccine efficacy and a low perceived risk of influenza infection [14]. In our study, only 62 % agreed that vaccination is the most effective way to prevent influenza, and only 16 % agreed that they get infected with influenza easily. In an analysis of HCP vaccination decisions, skewed risk perceptions concerning disease, transmission and vaccine side effects affected vaccination decisions. When the perceived risk of becoming infected is low, fear of side effects is the most frequent reason to decline vaccination [17]. In our study, most participants perceived themselves to be at low risk of infection with influenza, while a high proportion expressed concern over vaccine side effects.

In the multivariate analysis independent predictors of vaccination were perceived increased risk to self and family, and belief that vaccine is effective, while fear that the vaccine can cause disease was a barrier to vaccination. The belief that HCP might transmit influenza to patients was not independently associated with vaccine uptake. HCP are expected to be highly motivated to protect their patients. However, a review of studies on attitudes and predictors of HCP vaccination concluded that HCP get vaccinated primarily for their own benefit and not for the benefit of their patients [18]. A systematic review of factors associated with HCP acceptance of vaccination found that desire for self-protection and desire to protect family and friends increased acceptance, while concern that the vaccine causes the illness it was meant to prevent decreased acceptance [19].

In our study, almost all participants agreed that the risk of influenza transmission to patients is high. However, between these two motivators -- risk to self and risk to patients -- personal risk is the more powerful motivator of vaccine uptake.

Our study also showed that HCP who were vaccinated were more likely to recommend vaccination to patients and family.

Our study has several limitations. Volunteer bias may be present: 66 % of study participants indicated they were vaccinated as compared with the 42 % actual HCP vaccination rate, thus study participants represent HCP more inclined to vaccination. Since the survey was conducted after the influenza campaign, beliefs and attitudes may have been biased by behavior. There was also overrepresentation of allied health professionals. Since the study was anonymous, actual vaccination status of study participants could not be verified.

## Conclusions

Our findings suggest that HCP decline vaccination because they do not perceive a personal risk of influenza infection and are concerned about side effects. Preventing influenza transmission from HCP to patients, while most important from an administrative point of view, was not a powerful motivator. Thus, in order to increase vaccine coverage, it is important to continue educating HCP to correct misconceptions concerning vaccine safety and effectiveness while promoting the benefit of getting vaccinated in order to protect themselves and their family. We plan to use these findings in our influenza vaccination program and target populations with low vaccination rates for more intense intervention.
